# High Precision U/Th Dating of First Polynesian Settlement

**DOI:** 10.1371/journal.pone.0048769

**Published:** 2012-11-07

**Authors:** David Burley, Marshall I. Weisler, Jian-xin Zhao

**Affiliations:** 1 Department of Archaeology, Simon Fraser University, Burnaby, British Columbia, Canada; 2 School of Social Science, University of Queensland, St Lucia, Queensland, Australia; 3 Centre for Microscopy and Microanalysis, University of Queensland, St Lucia, Queensland, Australia; Universidad Autonoma de Barcelona and University of York, Spain

## Abstract

Previous studies document Nukuleka in the Kingdom of Tonga as a founder colony for first settlement of Polynesia by Lapita peoples. A limited number of radiocarbon dates are one line of evidence supporting this claim, but they cannot precisely establish when this event occurred, nor can they afford a detailed chronology for sequent occupation. High precision U/Th dates of *Acropora* coral files (abraders) from Nukuleka give unprecedented resolution, identifying the founder event by 2838±8 BP and documenting site development over the ensuing 250 years. The potential for dating error due to post depositional diagenetic alteration of ancient corals at Nukuleka also is addressed through sample preparation protocols and paired dates on spatially separated samples for individual specimens. *Acropora* coral files are widely distributed in Lapita sites across Oceania. U/Th dating of these artifacts provides unparalleled opportunities for greater precision and insight into the speed and timing of this final chapter in human settlement of the globe.

## Introduction

**Figure 1:**
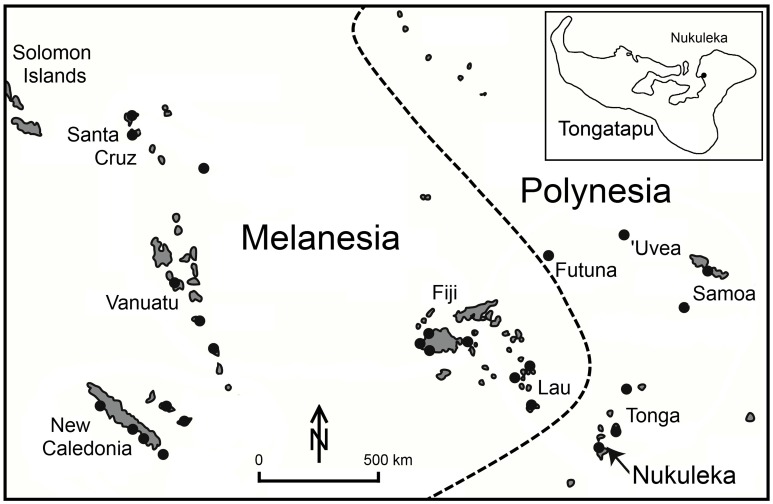
Lapita site distribution in Remote Oceania. Black dots represent Lapita sites or site concentrations. Nukuleka occurs on the southern island of Tongatapu, Kingdom of Tonga. The Nukuleka site map is provided as Figure S1.

The final chapter for human settlement of the globe began late in the 2nd millennium BC. Maritime adapted Austronesian-speaking groups from the Bismarck Archipelago off coastal New Guinea migrated eastward crossing into the uninhabited islands of central Oceania, ultimately reaching Tonga and then Samoa on the western flank of the Polynesian triangle (Figure 1). Collectively these groups are referred to as Lapita, after their distinctive and readily tracked ceramic industry [1]. The Lapita legacy is a significant one, with Lapita ancestry claimed by a large number of cultures and languages across the Pacific today, including Polynesian peoples. Over the past half century, a substantial volume of archaeological research has been dedicated to the Lapita migration and its implications [2]. One of the most fundamental concerns - a secure and precise chronology for the Lapita advance and its settlement history - has been a difficult task, given the need to find the earliest archaeological sites in different regions, and to acquire clearly associated and appropriate samples for radiocarbon dating. Recognized limitations of radiocarbon dating, including inbuilt age of wood samples, marine reservoir offsets and calibration into calendar years foster additional questions and debate.

Here we provide a precise chronology for first landfall in Polynesia, employing high precision U/Th dating of coral files from the Polynesian founder settlement at Nukuleka, Kingdom of Tonga [3], [4]. Coral files are rasp-like abraders of *Acropora* coral used to smooth, reduce or sculpt surfaces on wood or shell. Because they show wear related to human modification of live-collected coral fingers, their use and deposition resolves questions of indirect association often characteristic of radiocarbon samples [5]. U/Th dating also provides low standard errors in determination of late Holocene coral ages, exactness well beyond the capabilities of radiocarbon methods [6], [7], [8].

### Nukuleka: The Founder Colony for Polynesia

Nukuleka is located at the northeast entrance of the Fanga ‘Uta/Fanga Kakau lagoon system on the island of Tongatapu, Kingdom of Tonga (Figure 1). The Nukuleka locale is strategically positioned for access to open ocean as well as for inner reef fishing and marine foraging. This village has been occupied continuously since the initial Lapita settlement phase. Accumulated archaeological data identify Nukuleka as the earliest site in Tonga with expansion by later Lapita peoples occurring around the lagoon and then northward into the remaining islands of Tonga as well as Samoa [9]. Nukuleka represents a founder colony through which first settlement in Polynesia was funneled.

The status of Nukuleka as a founder colony is verified through four lines of evidence. First, while limited, Nukuleka radiocarbon dates are the earliest for any Lapita site in Polynesia (Table S1). Second, decorated ceramics from Nukuleka incorporate an assemblage of Lapita wares similar to those recovered from earlier Lapita sites in island Melanesia to the west of Tonga. These are markedly different from later Lapita ceramics in West Polynesia, and Nukuleka is the only site in West Polynesia where these early ceramics occur [9]. Third, a subset of the ceramic assemblage with the earliest Lapita designs is foreign to Tonga, based on petrographic analysis of ceramic temper sands and sherd geochemistry [4]. These pots were transported from the ancestral homeland of the Nukuleka colonizers, a homeland that has yet to be identified. And fourth, the settlement at Nukuleka expanded over a 20 ha area on the Nukuleka Peninsula during the 200–250 year period of Lapita occupation [9]. Nukuleka became a central place for Lapita peoples in West Polynesia as well as a gateway community for expanded settlement.

Archaeological excavations at Nukuleka have been concentrated in the back southwest corner of the contemporary village. Here Lapita ceramics as well as shellfish debris are scattered across the surface of a late (∼ 500 BP) prehistoric burial mound and in other exposures. Excavations into the mound reveal a Lapita kitchen midden immediately below [9], [10]. Mound construction fill was taken from adjacent midden deposits, resulting in an abundant and continuous distribution of material from the mound surface through the lowest cultural levels. *Acropora* coral file abraders, including the samples dated here, occur within this assemblage. Additional information on the Nukuleka site and its excavation is included in Supporting Information (Text S1).

Acquisition of a radiocarbon-dated chronology for Nukuleka has been a difficult process. Wood charcoal samples, other than very small flecks, rarely occur, and the degree of site disturbance from late prehistoric, historic and modern activities immediately raises issues of *in situ* stratigraphic association. Inbuilt age for unidentified species of wood charcoal creates additional uncertainty for radiocarbon dates [11], [12]. Radiocarbon dating of shellfish is problematic, requiring precise development of a marine reservoir correction (ΔR). This is made more complex for Nukuleka by the influence of “hard water effect” in the Fanga ‘Uta/Fanga Kakau lagoon system [13] and variability in marine reservoir offsets for different species of shellfish [14]. For Nukuleka only one AMS radiocarbon date (WK 23710) on charred nut addresses the inbuilt age issue, providing a 2σ 178-year calibrated range of 2769–2947 BP for initial site occupation (Table S1). Nukuleka radiocarbon dates are an inadequate data set to establish a precise age for first human landfall in Polynesia.

### Sample Context

Burley undertook excavations in 2007 at Nukuleka [9]. These were concentrated within the mound as well as in an area 45 m to the northwest of the mound (Figure S1). Ceramic and other artifacts from the mound area indicate a dominantly if not exclusively Lapita occupation; the area to the northwest has a sequence of occupation from the Lapita era through later prehistory. At the time of first landfall in Tonga, sea level on the Tongatapu lagoon is estimated 1.2 m higher than today [15] with the mound area being an active beach. As sea level fell and the coastal flat at Nukuleka expanded, the mound area became increasingly distant from the shoreline, and residential occupation was abandoned.

**Figure 2:**
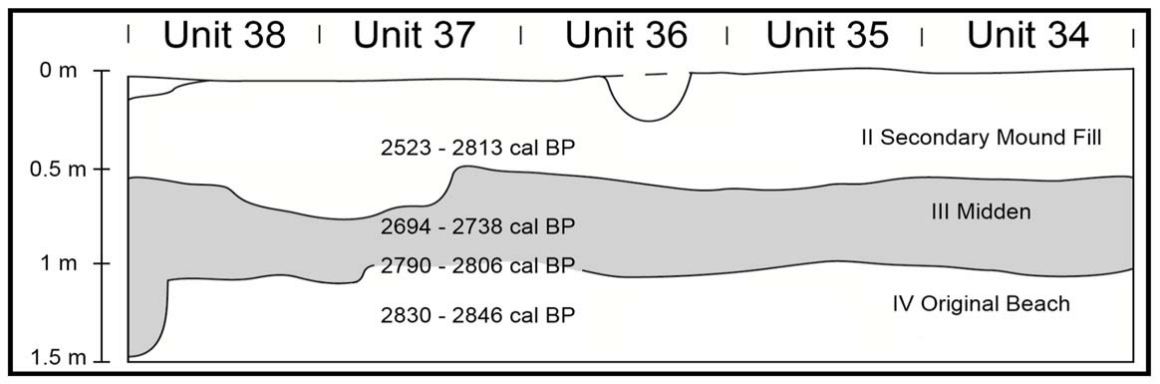
West stratigraphic profile of mound excavation with U/Th dates. Unit provenience is given in excavation plan Figure S1. Chronological intervals for Strata II to IV are based on minimum/maximum dates for U/Th date ranges within stratum as given in Table 1. There is no Stratum I in this part of the site.

Four principal strata were encountered in mound area excavations (Figure 2, Figure S2). Stratum I represents modern sediment deposition over parts of the mound closest to a back village road. Stratum II is a secondary deposit of burial mound fill from the adjacent Lapita midden. Stratum III is the *in situ* Lapita midden. Stratum IV is a calcareous yellow sand beach upon which the midden developed. Anthropogenic mixing is present in Stratum IV, as a limited number of early ceramic sherds and other artifacts are buried within this deposit [9]. Excavations to the northwest of the mound are located on a natural rise with cultural materials extending to a depth of 1.2 m. Stratigraphy here corresponds with cultural occupation levels, including upper a-ceramic occupations (Stratum I), a Polynesian Plainware ceramic phase occupation (Stratum II) as well as Lapita ceramic phase (Stratum III) deposits.

**Table 1:** Accepted U/Th dates and *Acropora* coral file stratigraphic associations.

U/Th Date	Area	Stratum	Depth	Lab #
2692±10	Mound	II	55–65	2011-030
2805±8	Mound	II	65–75	2011-034
2530±7	Mound	II	85–95	2011-037
2625±6	Mound	II	85–95	2011-020
2702±8	Mound	III	85–95	2011-032
2726±7	Mound	III	85–95	2011-023
2724±8	Mound	III	95–105	2011-022
2730±8	Mound	III	95–105	2011-029
2798±8	Mound	III/IV	105–115	2011-033
2838±8	Mound	IV	125–135	2011-036
2756±7	Northwest	II	75–85	2011-026
2704±6	Northwest	III	85–95	2011-025
2738±10	Northwest	III	85–95	2011-024

Provenience and measurement data for specimens are given in Table S2. U/Th isotopic data, including samples with alteration, are given in Table S3. Where paired sample dates are present (Table S3), the weighted mean is calculated. Age uncertainties are given at a 2σ level.

**Figure 3:**
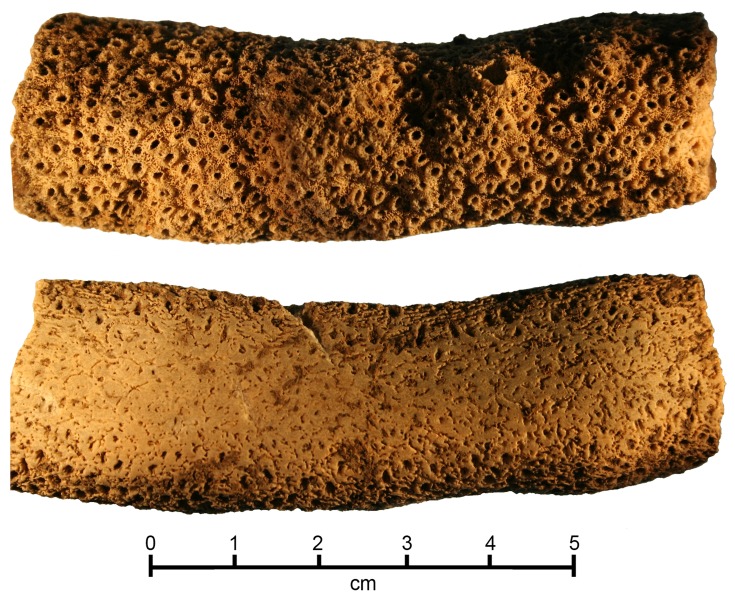
Pristine (upper) and used (lower) surfaces of an *Acropora* coral file (Lab # 024) dated for this study. Note the fine sculptural details on the unworn surface.

**Figure 4:**
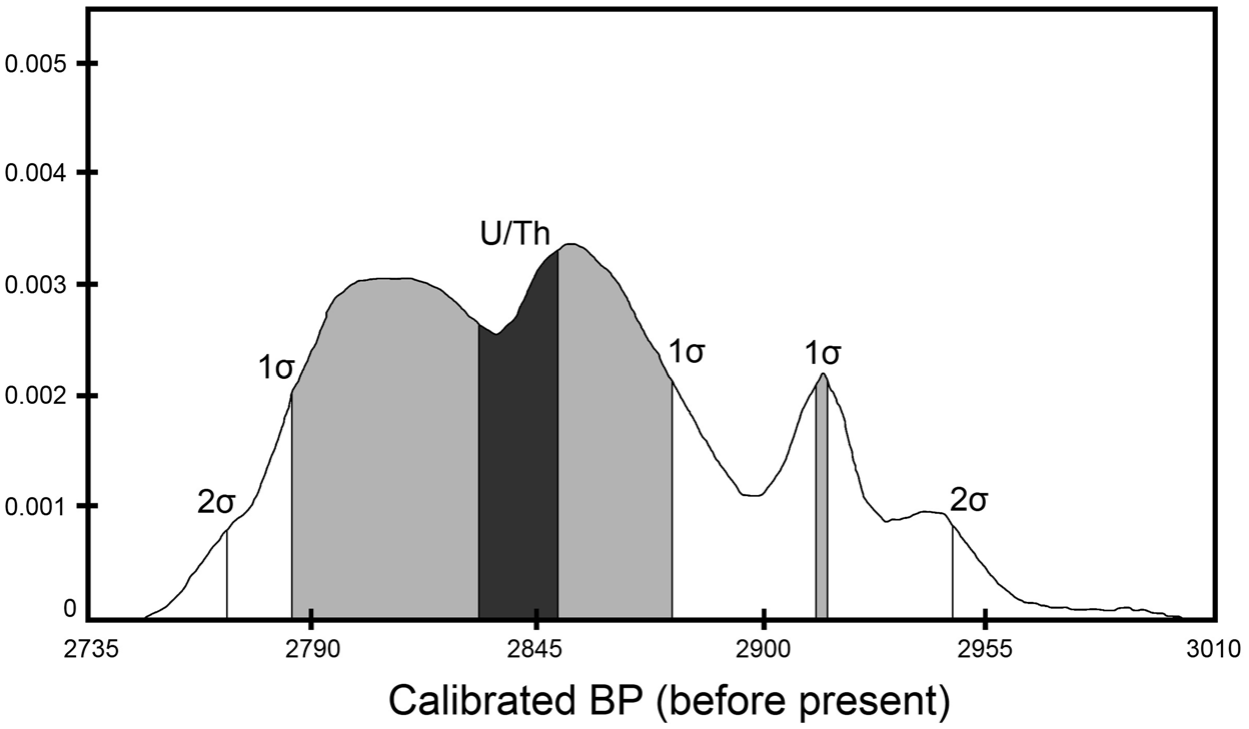
Plot of relative probabilities of 1σ (light shading) and 2σ calibrations for ^14^C date WK 23710 (2811±35) from Stratum IV at Nukuleka. Darkened area is U/Th date range for sample 2011-036 (2838±8) from Stratum IV. WK 23710 is taken from a short-lived charred nut. The radiocarbon calibration was carried out using Calib 5.1 radiocarbon calibration program employing the southern hemisphere 2004 calibration curve [21].

## Materials and Methods

### Ethics Statement

All archaeological coral samples analysed in the study were collected under a Research Permit (2003–2008) issued to DVB by the Prime Ministers Office, Government of Tonga, Nuku’alofa, Tonga.

A total of 16 coral files were considered appropriate for U/Th dating from the 2007 excavations. All specimens appear to be the same species of a large staghorn-like form of *Acropora* coral commonly found on reef slopes and lagoons [16]. It is assumed that files having well-preserved and sharp corallites were originally harvested live from the reef. All files have abraded flat surfaces indicative of use (Figure 3).

Previous U/Th dating of corals in Pacific archaeology has been conducted on more recent samples, virtually all less than 500 years old [6], [7], [8]. Nukuleka samples are considerably older, enhancing possibilities for post-depositional diagenetic alteration with potential impact on U/Th dates. To assess/accommodate alteration, coral files were sectioned perpendicular to the growth axis (Figure S3). Two samples were considered pristine without further concern. For the remainder, two adjacent 1 cm long sections closest to the tip were taken for dating. Annual growth rates of branching *Acropora* corals is typically 70–330 mm/yr [17], meaning the age difference between samples should be less than one year. Samples were broken into grains of 1–2 mm diameter and pretreated as described in Text S2. Samples were then handpicked for the cleanest/pristine grains with 100 mg subsamples acquired. Subsamples were dated on a Nu Plasma multi-collector inductively coupled plasma mass spectrometer (MC-ICP-MS) in the Radiogenic Isotope Facility, the University of Queensland. Analytic methods follow those reported in Zhao et al. [18] and Zhou et al. [19]. If dates for sample pairs differ substantially beyond their 2σ range, we assume diagenesis is present and the dates are considered unreliable. This was the case for three of the 16 coral files being analyzed. Where the measured ^230^Th ages for Sections A and B are within their 2σ errors, the weighted mean has been calculated as presented in Table 1 and Table S3. All age uncertainties are given at the 2σ level.

## Results

Corrected U/Th dates for the 13 coral file samples without alteration as well as provenience and stratigraphic data are provided in Table 1. Detailed analytic data, including those for the three unreliable dates, are provided in Table S3 while additional sample information is given in Table S2. Ten of the accepted U/Th dated samples come from mound excavations of Strata II (n = 4), III (n = 4), IV (n = 1) and the Stratum III/IV transitional break (n = 1). As a group, this series of dates is significantly robust (Table 1, Figure 2). The single sample from Strata IV yields a date of 2838±8 BP, representing the earliest sample from mound area excavations. It is associated with a small assemblage of early Lapita ceramics, including a number of the foreign sherds tied to first landfall. A single date of 2798±8 BP comes from a coral file at the interface of Stratum III with IV. This dates the beginning of intensive development of midden deposits with shell, organics and other materials. The four Stratum III dates provide a range of 2738-2694 BP for these deposits. *In situ* proveniences for these samples are consistent; the earliest coral file date in Stratum III (2730±8 BP) occurs lower in the stratum than the most recent dated sample (2702±8 BP). The remaining dates from Stratum II have no *in situ* provenience integrity, since they were derived from secondary mound fill. With one exception, these dates correspond with the Lapita era temporal interval for Tonga, and they confirm ceramic-based interpretations of the mound area midden as dominantly Lapita in composition [9]. The exception is 2530±7 BP, a date falling within chronological expectations of the early Polynesian Plainware phase (2650-1600 BP) in Tonga [20].

The remaining three accepted U/Th coral file dates come from the northwest excavation. Two of these were recovered from Stratum III Lapita age deposits while the other was associated with Stratum II, a later Polynesian Plainware occupation. A date of 2756±7 BP for the latter specimen is earlier than the hypothesized cultural association. It is probable that the sample is out of context due to stratigraphic disturbance [9](Text S1). The two other samples come from the upper part of Stratum III from a provenience believed to have stratigraphic integrity. Coral file dates of 2738±10 and 2704±6 BP correspond exactly with hypothetical expectations.

## Discussion

Stratum IV below the Lapita midden and late prehistoric burial mound is an original beach surface. Artifacts within this stratum are explainable only as a result of loss and trampling by initial site occupants, perhaps combined with high-tide wash-over with additional coral sand deposition. The limited number of artifacts suggests a brief period of time before accumulating midden debris sealed the stratum. Early Lapita ceramics with temper and pastes foreign to Tonga occur within the stratum, associating it with first landfall in Polynesia. The Stratum IV coral file date of 2838±8 BP provides a very precise age for this event. We feel additionally secure in this interpretation through comparison of the coral file date with the only AMS radiocarbon date from Stratum IV based on a short-lived wood species. When the coral file range is plotted against the calibrated range for the radiocarbon date, it falls virtually in the middle of the 1σ probability (Figure 4). This comparison further illustrates the significant differences in precision between U/Th dates on coral files, and those based on even the best samples for AMS radiocarbon dating.

The remaining series of coral dates, whether in primary or secondary context, provide additional insight into site formation and land use at Nukuleka. The Stratum III/IV date of 2798±8 BP indicates a period of 30 or so years between early occupation on the beach and the beginnings of midden accumulation. All but one of the mound area dates is of Lapita-age indicating an almost exclusive Lapita occupation in this part of the site. And the most recent date for the mound area of 2530±7 BP provides a *terminus post quem* (date after which) for residential abandonment of the mound area resulting from sea level fall and development of the Nukuleka coastal flat.

### Conclusion

The stratigraphic coherency of dates at Nukuleka demonstrates the efficacy of high precision U/Th dating of Lapita-age coral files to narrowly constrain occupation history. They also indicate a 16-year interval of 2830–2846 BP for first human landfall in Polynesia. This precision is far greater than is possible by radiocarbon measurement, even when charcoal samples come from short-lived species. Coral files, as those employed here, are widely distributed in Lapita sites from the Bismarck Archipelago into western Polynesia. With appropriate protocols for diagenetic alteration, our ability to precisely date these sites by U/Th assay provides unparalleled opportunities for gaining new insights into the final chapters of human settlement of the globe.

## Supporting Information

Figure S1 **Nukuleka Site Excavation Map.** Nukuleka site excavations (darkened blocks) and mound perimeter (dark dashes). The north/south trench through the mound was excavated in 1965 [1] while the remainder was carried out by Burley [5]. Excavation unit numbers cross reference with Table S3. Stippled areas are roads, dashed lines represent fences and hatched rectangles are structures.
(TIF)

Figure S2 **Nukuleka Excavation Stratigraphy.** Block excavation north face stratigraphy (see Figure S1 for location). Stratum III occurs only in the west half of this section. Stratum IV is the yellow coral sand beach beneath the midden deposit. Stratum III/IV is the interface between the beach and midden.
(TIF)

Figure S3 **Sample Sections and Cleaning.** Photos showing sample cleaning steps and representative samples used for U/Th dating. See Text S2 for details. Note the differences between pristine sample 2011-024 (upper left) and the darker sample 2011-027 (lower left) resulting from diagenetic alteration.
(TIF)

Table S1 **Radiocarbon Dates for Nukuleka.** Radiocarbon dates for Nukuleka [5]. For ANU 541, Spennemann and Head [18] employ a lagoon specific reservoir correction to provide a corrected date of 2819±89 BP and a calibration range as given. Calibration for the remainder is carried out using the Calib 5.1 radiocarbon calibration program employing the southern hemisphere 2004 calibration curve [19]. All dates except for ANU 541 are AMS measurements. Samples identified as wood charcoal typically are small flecks and have not been identified to species.
(DOCX)

Table S2 **U/Th Sample Proveniences and Metric Data.** Provenience and metric data for U/Th samples without alteration. Samples are ordered by depth within stratigraphic units. Upper 11 samples come from mound area excavations. Stratum II in the mound area is secondary fill deposit without stratigraphic integrity. Bottom three units come from the northwest excavation area. Sample 2011-026 from Stratum II Unit 57 has an out of context U/Th date probably resulting from stratigraphic disturbance. Figure S1 locates numbered excavation units at Nukuleka.
(DOCX)

Table S3 **U/Th isotopic data for** ***Acropora*** **coral file samples.** U/Th isotopic data for *Acropora* coral file samples from Nukuleka. Ratios in parentheses are activity ratios calculated from the atomic ratios. The ages were calculated using Isoplot EX 3.0 program [12] with decay constants from Cheng et al [13]. Corr. and uncorr. denote corrected and uncorrected. The corrected ^230^Th ages were corrected for initial ^230^Th using an assumed bulk-Earth atomic ^230^Th/^232^Th atomic ratio of 4.4±2.2×10^−6^. The age uncertainties for the weighted mean age are at 2σ.
(PDF)

Text S1 **Study Site Excavations and U/Th Dating Methods.**
(DOCX)
