# Supplementary figures and images for: High Precision U/Th Dating of First Polynesian Settlement
Source: PLoS One. 2012 Nov 7;7(11):e48769. doi: 10.1371/journal.pone.0048769 (PMC3492438; doi:10.1371/journal.pone.0048769)

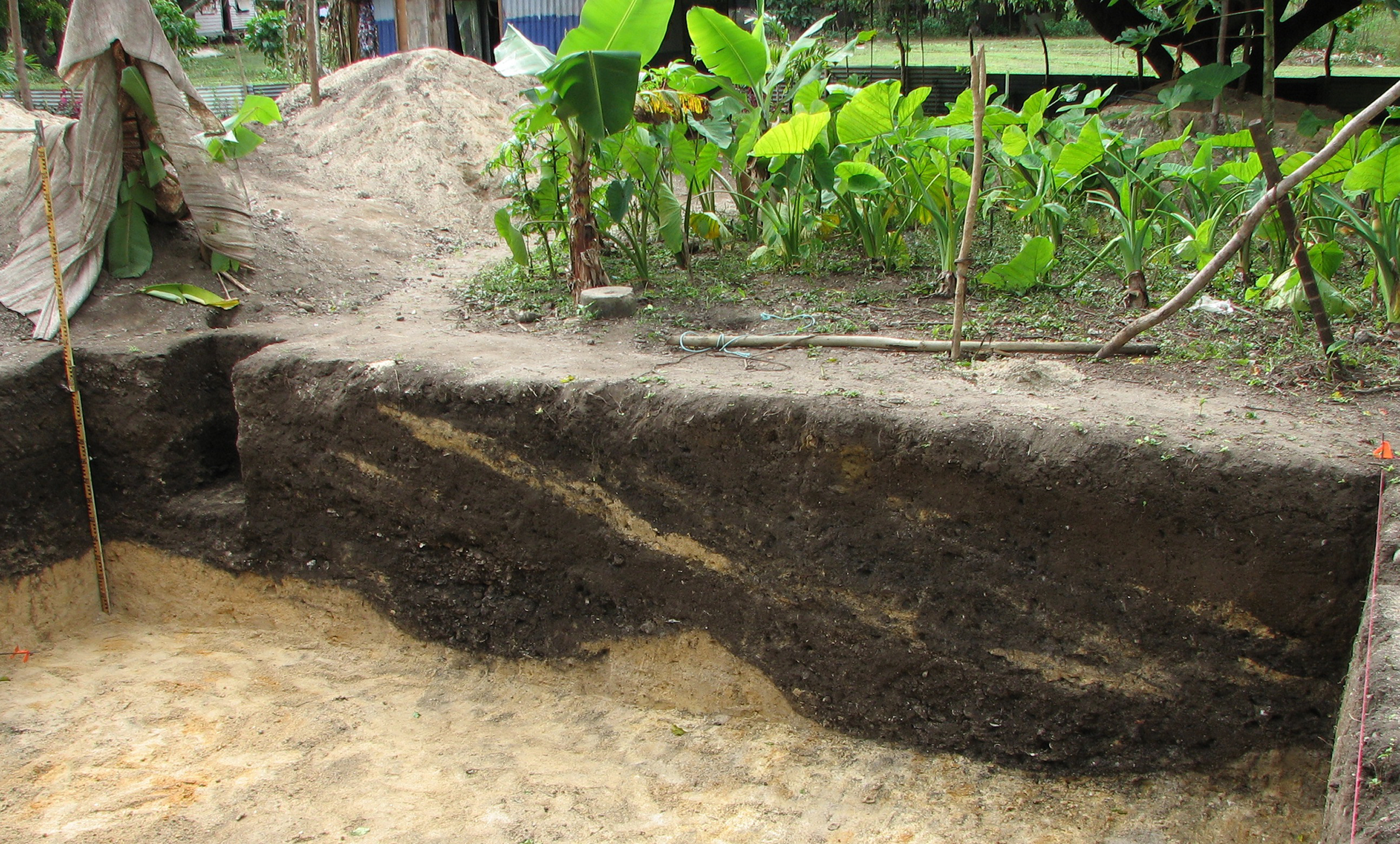

Supplement: Figure S2 — Nukuleka Excavation Stratigraphy. Block excavation north face stratigraphy (see Figure S1 for location). Stratum III occurs only in the west half of this section. Stratum IV is the yellow coral sand beach beneath the midden deposit. Stratum III/IV is the interface between the beach and midden. (TIF) [file pone.0048769.s002.tif]

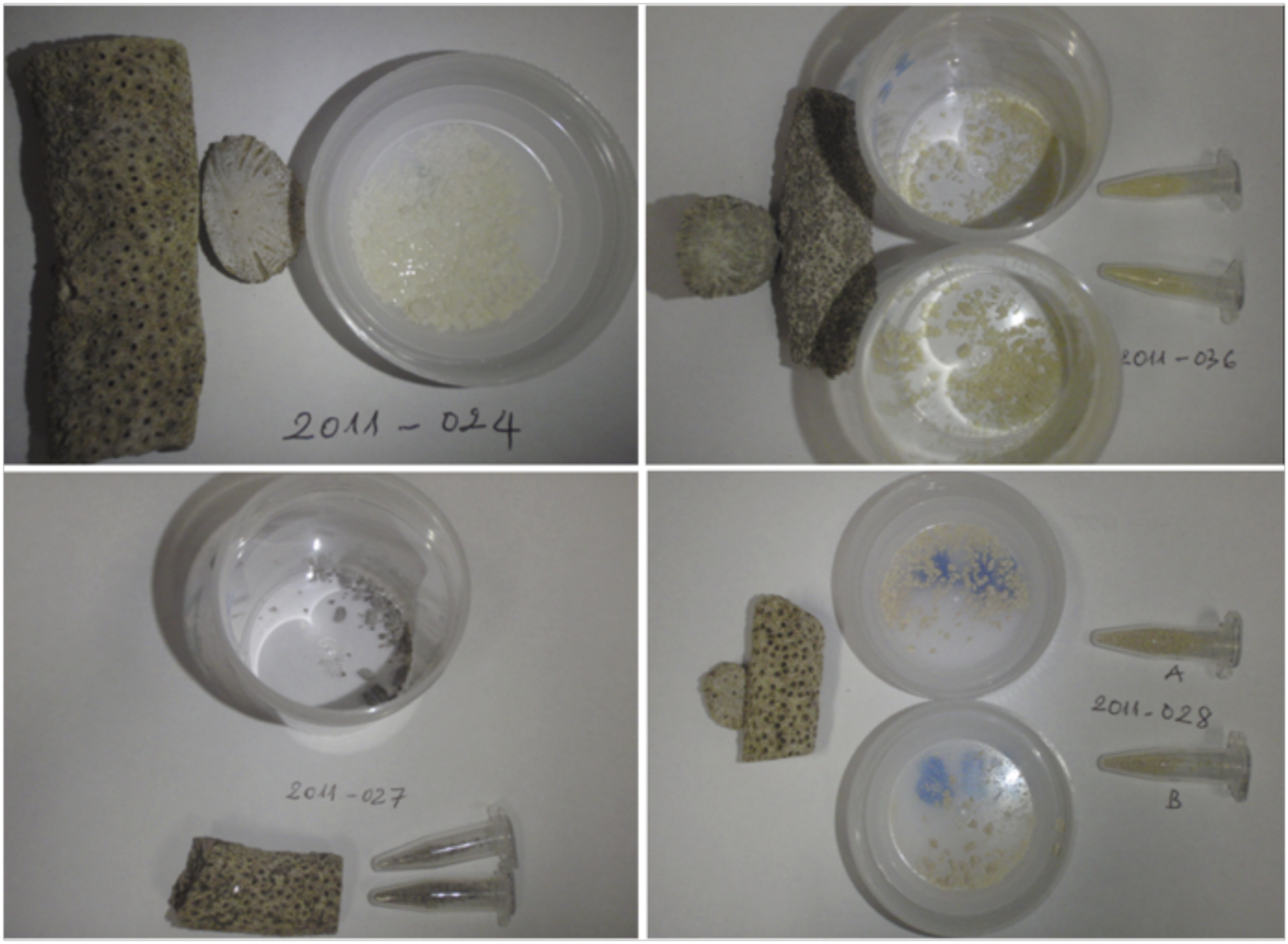

Supplement: Figure S3 — Sample Sections and Cleaning. Photos showing sample cleaning steps and representative samples used for U/Th dating. See Text S2 for details. Note the differences between pristine sample 2011-024 (upper left) and the darker sample 2011-027 (lower left) resulting from diagenetic alteration. (TIF) [file pone.0048769.s003.tif]
