# Supplementary material for: High Precision U/Th Dating of First Polynesian Settlement
Source: PLoS One. 2012 Nov 7;7(11):e48769. doi: 10.1371/journal.pone.0048769 (PMC3492438; doi:10.1371/journal.pone.0048769)
Supplement: Table S1 — Radiocarbon Dates for Nukuleka. Radiocarbon dates for Nukuleka [5]. For ANU 541, Spennemann and Head [18] employ a lagoon specific reservoir correction to provide a corrected date of 2819±89 BP and a calibration range as given. Calibration for the remainder is carried out using the Calib 5.1 radiocarbon calibration program employing the southern hemisphere 2004 calibration curve [19]. All dates except for ANU 541 are AMS measurements. Samples identified as wood charcoal typically are small flecks and have not been identified to species. (DOCX) [file pone.0048769.s004.docx]

| Lab Number | Material | Date | δ^13^C | Calibrated 2σ | Stratum |
| --- | --- | --- | --- | --- | --- |
|  |  |  |  |  |  |
| ANU 541 | marine shell | 3090±95 | unknown | 2781-3026 BP | III/IV? |
| WK 23708 | wood charcoal | 2836±32 | -25.9 | 2781-2963 BP | IV |
| WK 23710 | charred nut | 2811±35 | -23.5 | 2769-2947 BP | IV |
| CAMS 59624 | wood charcoal | 2790±50 | -24.6 | 2753-2949 BP | III/IV |
| WK 23707 | wood charcoal | 2696±32 | -24.5 | 2721-2844 BP | IV |
| WK 23709 | wood charcoal | 2536±32 | -24.6 | 2364-2718 BP | III |
