# Supplementary material for: High Precision U/Th Dating of First Polynesian Settlement
Source: PLoS One. 2012 Nov 7;7(11):e48769. doi: 10.1371/journal.pone.0048769 (PMC3492438; doi:10.1371/journal.pone.0048769)
Supplement: Table S2 — U/Th Sample Proveniences and Metric Data. Provenience and metric data for U/Th samples without alteration. Samples are ordered by depth within stratigraphic units. Upper 11 samples come from mound area excavations. Stratum II in the mound area is secondary fill deposit without stratigraphic integrity. Bottom three units come from the northwest excavation area. Sample 2011-026 from Stratum II Unit 57 has an out of context U/Th date probably resulting from stratigraphic disturbance. Figure S1 locates numbered excavation units at Nukuleka. (DOCX) [file pone.0048769.s005.docx]

| **Lab #** | **Cat #** | **Unit** | **Spit** | **Depth** | **Stratum** | **U/Th Date** | **Wt** | **Length** | **Width** | **Thick** |
| --- | --- | --- | --- | --- | --- | --- | --- | --- | --- | --- |
| 2011-030 | 109 | 11 | 5 | 55-65 | II | 2692±10 | 54 | 108.26 | 19.08 | 15.84 |
| 2011-034 | 219 | 16 | 6 | 65-75 | II | 2805±8 | 25.2 | 67.09 | 18.6 | 14.82 |
| 2011-037 | 295 | 20 | 8 | 85-95 | II | 2530±7 | 31.4 | 50.4 | 20.29 | 16.78 |
| 2011-020 | 412 | 27 | 8 | 85-95 | II | 2625±6 | 39.6 | 104.35 | 18.9 | 10.71 |
| 2011-032 | 204 | 15 | 8 | 85-95 | III | 2702±8 | 33.5 | 86.48 | 17.26 | 9.11 |
| 2011-023 | 521 | 36 | 8 | 85-95 | III | 2726±7 | 164.7 | 86.25 | 35.83 | 32.49 |
| 2011-022 | 498 | 34 | 9 | 95-105 | III | 2724±8 | 11.1 | 51.54 | 16.48 | 10.41 |
| 2011-029 | 90 | 10 | 9 | 95-105 | III | 2730±8 | 143.4 | 123.71 | 28.08 | 23.24 |
| 2011-033 | 209 | 15 | 10 | 105-115 | III/IV | 2798±8 | 74.3 | 85.8 | 23.35 | 19.61 |
| 2011-036 | 290 | 19 | 12 | 125-135 | IV | 2838±8 | 69.5 | 116.87 | 20.33 | 16.69 |
| 2011-026 | 602 | 57 | 8 | 75-85 | II | 2756±7 | 26.7 | 78.86 | 15.7 | 10.07 |
| 2011-025 | 568 | 56 | 9 | 85-95 | III | 2704±6 | 74.6 | 94.64 | 23.7 | 15.37 |
| 2011-024 | 569 | 56 | 9 | 85-95 | III | 2738±10 | 45.4 | 77.5 | 22.23 | 14.37 |
