# Supplementary material for: High Precision U/Th Dating of First Polynesian Settlement
Source: PLoS One. 2012 Nov 7;7(11):e48769. doi: 10.1371/journal.pone.0048769 (PMC3492438; doi:10.1371/journal.pone.0048769)
Supplement: Table S3 — U/Th isotopic data for Acropora coral file samples. U/Th isotopic data for Acropora coral file samples from Nukuleka. Ratios in parentheses are activity ratios calculated from the atomic ratios. The ages were calculated using Isoplot EX 3.0 program [12] with decay constants from Cheng et al [13]. Corr. and uncorr. denote corrected and uncorrected. The corrected 230Th ages were corrected for initial 230Th using an assumed bulk-Earth atomic 230Th/232Th atomic ratio of 4.4±2.2×10^−6^. The age uncertainties for the weighted mean age are at 2σ. (PDF) [file pone.0048769.s006.pdf]

Sample Name	U (ppm)	±2σ	^{232}Th (ppb)	±2σ	(^{230}Th/ ^{232}Th)	±2σ	(^{230}Th/ ^{238}U)	±2σ	(^{234}U/ ^{238}U)	±2σ	uncorr. ^{230}Th Age (yr)	±2σ	corr. ^{230}Th Age (yr)	±2σ	corr. ^{230}Th Age (BP)	±2σ	Initial (^{234}U/ ^{238}U)	±2σ
2011-020A	3.6909	0.0008	0.0189	0.0005	16417	396	0.02773	0.00010	1.1446	0.0011	2677	10	2677	10	2615	10	1.1457	0.0012
2011-020B	3.7126	0.0009	0.0197	0.0004	15960	322	0.02787	0.00007	1.1442	0.0011	2692	8	2692	8	2630	8	1.1453	0.0011
2011-020 mean															2625	6		
2011-021A	3.5106	0.0008	0.0190	0.0004	16211	349	0.02893	0.00012	1.1443	0.0009	2795	12	2795	12	2733	12	1.1454	0.0009
2011-021B	3.5482	0.0010	0.0093	0.0007	34944	2582	0.03018	0.00015	1.1454	0.0011	2914	14	2914	14	2852	14	1.1466	0.0012
2011-021 mean															**2793**	**13**		
2011-022A	3.6128	0.0011	0.0178	0.0005	17733	530	0.02882	0.00010	1.1448	0.0013	2783	10	2782	10	2721	10	1.1459	0.0013
2011-022B	3.6159	0.0008	0.0136	0.0004	23353	685	0.02887	0.00011	1.1446	0.0014	2789	11	2789	11	2727	11	1.1457	0.0014
2011-022 mean															2724	8		
2011-023A	3.6211	0.0008	0.0002	0.0000	1986695	124602	0.02875	0.00009	1.1436	0.0012	2779	9	2779	9	2717	9	1.1447	0.0012
2011-023B	3.5041	0.0010	0.0077	0.0002	39903	1269	0.02898	0.00010	1.1454	0.0009	2797	10	2797	10	2735	10	1.1465	0.0009
2011-023 mean															2726	7		
2011-024	3.8078	0.0012	0.0062	0.0002	54019	1616	0.02899	0.00010	1.1446	0.0010	2800	10	2800	10	2738	10	1.1457	0.0010
2011-025A	3.7899	0.0011	0.0038	0.0002	87887	3936	0.02872	0.00007	1.1449	0.0009	2772	8	2772	8	2711	8	1.1460	0.0009
2011-025B	3.7523	0.0011	0.0068	0.0002	48143	1612	0.02858	0.00008	1.1454	0.0014	2758	9	2758	9	2696	9	1.1466	0.0014
2011-025 mean															2704	6		
2011-026A	3.3312	0.0008	0.0196	0.0004	15013	286	0.02915	0.00012	1.1450	0.0011	2815	12	2814	12	2753	12	1.1462	0.0011
2011-026B	3.3507	0.0006	0.0277	0.0003	10724	130	0.02922	0.00009	1.1454	0.0012	2821	10	2820	10	2759	10	1.1465	0.0012
2011-026 mean															2756	7		
2011-027A	3.1054	0.0009	0.8847	0.0009	355	1	0.03337	0.00011	1.1453	0.0014	3227	12	3220	12	3165	12	1.1466	0.0014
2011-027B	2.9837	0.0011	1.3719	0.0014	236	1	0.03580	0.00009	1.1469	0.0010	3461	9	3449	11	3399	11	1.1483	0.0010
2011-027 mean															**3282**	**12**		
2011-028A	3.8986	0.0008	0.0881	0.0005	3824	23	0.02846	0.00008	1.1445	0.0012	2749	9	2748	9	2687	9	1.1456	0.0012
2011-028B	4.4989	0.0018	0.0135	0.0004	30472	946	0.03009	0.00009	1.1461	0.0010	2903	10	2903	10	2842	10	1.1473	0.0010
2011-028 mean															**2764**	**9**		
2011-029A	4.1175	0.0023	0.0064	0.0002	56497	2017	0.02883	0.00010	1.1439	0.0009	2786	10	2786	10	2725	10	1.1450	0.0009
2011-029B	3.2376	0.0009	0.0096	0.0003	29595	918	0.02898	0.00012	1.1444	0.0010	2799	12	2799	12	2737	12	1.1455	0.0010
2011-029 mean															2730	8		
2011-030	3.2208	0.0008	0.0771	0.0008	3620	39	0.02855	0.00010	1.1463	0.0009	2753	10	2753	10	2692	10	1.1474	0.0009
2011-032A	3.5264	0.0007	0.0022	0.0001	69152	4275	0.02865	0.00012	1.1457	0.0009	2763	12	2763	12	2702	12	1.1469	0.0009
2011-032B	3.5935	0.0007	0.0059	0.0003	52576	2336	0.02862	0.00011	1.1447	0.0011	2763	11	2763	11	2701	11	1.1459	0.0011
2011-032 mean															2702	8		
2011-033A	4.2523	0.0010	0.0184	0.0011	20811	1188	0.02974	0.00014	1.1450	0.0011	2873	14	2872	14	2811	14	1.1462	0.0011
2011-033B	4.0647	0.0010	0.0116	0.0003	31523	756	0.02953	0.00010	1.1444	0.0009	2853	10	2853	10	2791	10	1.1456	0.0009
2011-033 mean															2798	8		
2011-034A	4.4772	0.0011	0.0150	0.0006	27121	1098	0.02987	0.00012	1.1454	0.0009	2884	12	2884	12	2822	12	1.1466	0.0009
2011-034B	4.4503	0.0009	0.0218	0.0005	18270	383	0.02953	0.00012	1.1458	0.0010	2849	12	2849	12	2788	12	1.1470	0.0010
2011-034 mean															2805	8		
2011-036A	4.7577	0.0011	0.0288	0.0006	15029	330	0.02994	0.00012	1.1450	0.0006	2892	12	2891	12	2830	12	1.1462	0.0006
2011-036B	4.8567	0.0014	0.0306	0.0006	14517	288	0.03012	0.00011	1.1460	0.0011	2907	11	2907	11	2845	11	1.1472	0.0011
2011-036 mean															2838	8		
2011-037A	5.2961	0.0013	0.0106	0.0005	40851	1888	0.02699	0.00011	1.1452	0.0009	2603	11	2603	11	2541	11	1.1462	0.0010
2011-037B	5.3041	0.0015	0.0122	0.0006	35327	1706	0.02680	0.00010	1.1460	0.0011	2583	10	2583	10	2521	10	1.1471	0.0011
2011-037 mean															2530	7		
