# Supplementary material for: High Precision U/Th Dating of First Polynesian Settlement
Source: PLoS One. 2012 Nov 7;7(11):e48769. doi: 10.1371/journal.pone.0048769 (PMC3492438; doi:10.1371/journal.pone.0048769)
Supplement: Text S1 — Study Site Excavations and U/Th Dating Methods. (DOCX) [file pone.0048769.s007.docx]

**Text S1. Study Site Excavations and U/Th Dating Methods**

**Study Site and Excavations**

The Nukuleka Lapita site was documented first by Poulsen [1] who conducted excavations here as well as at other Lapita sites on the island of Tongatapu in 1964 and 1965. Beginning in 1990 and continuing into present, Burley [2,3] carried out systematic archaeological survey for other Lapita settlements across Tonga, with extensive coverage in the central islands of Ha’apai, the northern islands of Vava’u and around the lagoon shore on Tongatapu. These surveys as well as excavations at 16 plus sites by Burley and others [4] indicate clearly the uniqueness of the Nukuleka Lapita ceramic assemblage. Burley returned to Nukuleka in 2007 for additional excavation as well as to document the expansive nature of this site across the Nukuleka Peninsula. These excavations solidify identification of Nukuleka as a Polynesian founder colony.

A detailed account of the 2007 excavation objectives, methods, as well as results is given in Burley et al. [5]. For the late prehistoric burial mound discussed in the article, an initial 3 x 4 m block excavation was expanded to 6 x 5 m with each 1 x 1 m unit given a numeric designation (Figure S1). Excavation was conducted by trowel, proceeding across the excavation area in tandem. Clear breaks were present between each of the stratigraphic units but internal stratigraphy in Strata III and IV was not discerned. Vertical provenience control was maintained through 10 cm arbitrary spits. Overall stratigraphy in the mound area block was complex owing to the processes of mound construction (Figure S2). Construction fill for the mound had been excavated from the eastern half of the block and thrown inward toward the mound centre. The Strata III midden, thus, occurs only in the western half of the excavation units. The *in situ* midden is a black loam with high organic content, shell, fire broken rock, abundant ceramics and faunal remains. Other 2007 excavations at the Nukuleka site include the 2 x 2 m block 45 m to the northwest as well as two 1 x 1 m units north of the mound (Figure S1). Stratigraphy in the northwest units was partially disturbed due to later pit and other feature excavations. Unit 56 is identified as having the greatest stratigraphic integrity [5].

The 2007 excavations recovered 47,527 ceramic sherds as well as 699 other artifacts. Among the latter are 60 coral files, with 50 of these coming from the mound area. Beads, bracelet fragments and other items of personal adornment dominate the non-ceramic artifact assemblage at Nukuleka. We believe the coral files may have been employed in the manufacture of these items as well as for other tasks. All 16 of the coral files selected for U/Th dating had unequivocal use as an abrader but also had segments of their surface with preserved and sharp corallites. Only one of the files was judged to be whole, measuring 123.71 mm long and 30.82 mm. Descriptive measurements and provenience information for unaltered specimens are provided in Table S2.

**U/Th Dating Method**

As described in Materials and Methods, each coral file was sectioned perpendicular to the growth axis (Figure S3). For all but two of the most pristine samples, two short sections about 1 cm in length next to each other in growth position, and closest to the tip, were taken and broken into small grains of 1-2 mm in diameter, respectively. As the typical annual growth rate of branching *Acropora* corals is usually 70-330 mm/yr [6], the age difference between the two small sections should be less than one year. The grained sub-samples were then soaked and ultra-sonicated in 30% H_2_O_2_ to break up any trace amount of organics adhering to the grains. This treatment proved to be very effective in removing organic matter, with treated grains being much fresher looking than the original coral files (Figure S3). Sub-samples were then washed ultrasonically in milli-Q water and air-dried in the oven at 60ºC. Once dry, the cleanest grains were handpicked and transferred into a small 0.6ml vial. Approximately 100 mg of carefully cleaned material from each sub-sample was used for U-series dating on a Nu Plasma multi-collector inductively coupled mass spectrometer (MC-ICP-MS) at the Radiogenic Isotope Facility (RIF), the University of Queensland.

Each sub-sample was spiked with a ^229^Th-^233^U mixed tracer and dissolved in double-distilled nitric acid. After complete digestion, a few drops of H_2_O_2_ were added, and the sample beakers tightly closed and placed on a hotplate at 90°C overnight to decompose any trace amounts of organic matter and to ensure complete tracer-sample mixing. After standard Fe hydroxide co-precipitation to pre-concentrate U and Th, the precipitates were re-dissolved in 0.2 ml 7N double-distilled HNO_3_ and purified using a simplified ion-exchange procedure modified after Zhao et al. [7]. After chemical separation, the Th fraction was mixed with a small percentage of U (sufficient to achieve 3-4 volts of ^238^U signal) and the mixture was diluted to 3-ml in 2% HNO_3_. The U/Th mixed solution was then injected into the MC-ICP-MS through a DSN-100 desolvation system with an uptake rate of around 0.12 ml per minute. U/Th isotopic ratio measurement was performed on the Nu Plasma MC-ICP-MS following the analytical protocol described by Hellstrom [8] with minor modifications to the detector configuration (e.g. the ^235^U on IC0 sequence of Hellstrom was not used in our protocol, instead we used ^233^U for IC0/Faraday gain calibration). The natural ^238^U/^235^U value of 137.88 was used for mass fractionation correction for both U and Th isotopic ratio measurements. The second electron multiplier IC2 was filtered with a fitted retardation lens to increase the abundance sensitivity by ten fold. This minimizes a large tailing of the ^232^Th signal into the ^230^Th peak, which is potentially very serious for impure carbonates with high ^232^Th/^230^Th. The working abundance sensitivity on IC2 is <500 ppb at one mass distance from the large peak in the U/Th mass range, thus tailing from ^232^Th to mass 230 is typically <1% of ^230^Th peak for the samples with ^232^Th/^230^Th atomic ratio <10,000. The tailing contribution was extracted from the ^230^Th signal by simultaneous measurements of baselines at masses 230.5 and 229.5. In fact, the ^232^Th/^230^Th atomic ratio in most samples in this study is typically <100, thus the tailing effect is completely negligible. The working sensitivity of the instrument is about 0.5-0.6 volt per ppb for U and 0.45-0.55 volt per ppb of Th. Each sample took about 20 minutes to measure. In between samples, a vigorous 15-minute cleaning procedure was undertaken and all isotopes were monitored and raw counts measured on their respective detectors to ensure no carry-over memories from previous samples. Measurements of samples, standards, and carryover memories were performed fully automatically using a CETAC ASX-110 auto-sampler. A long-term monitoring of the carryover memories over 15 months shows that ^230^Th memory is consistently less than 0.1 counts per second, which is negligible for all samples in this study. The memories for all other isotopes are also negligible. The procedural blanks for our MC-ICP-MS U/Th dating protocol are considerably smaller than those of our TIMS protocol reported in Weisler et al. [9] and Clark et al. [10], with mean measured ^238^U, ^232^Th and ^230^Th blanks at 1.6x10^-5^, 1.7x10^-5^ and 2.5x10^-10^ nmol (or 3.7 pg, 3.8 pg, 0.06 fg) respectively. ^230^Th blank extractions for our samples of ~0.1 g in size correspond to only about one year, which is also negligible. A standard solution of the secular equilibrium Harwell Uraninite, HU-1 (which was used for our spike calibration, see Zhao et al. [11]) and an in-house purpose-made spiked solution (its isotopic compositions were precisely calibrated against the HU-1 standard) were analysed repeatedly in between every 5-6 unknown samples during each fully-automated session to calculate the long-term reproducibility. This long-term monitoring shows that the external 2σ errors for repeated ^230^Th/^238^U and ^234^U/^238^U measurements are only ~1.3 and ~1.5 times larger than their average internal 2σ errors. Therefore internal 2σ errors of ^230^Th/^238^U and ^234^U/^238^U measurements of the unknown samples were magnified 1.3 and 1.5 times, respectively. Such a slight error magnification, which is typical of isotopic measurements by mass spectrometry, is related to the cumulative errors derived from imperfect peak shapes and detector alignments, imperfect peak centering, errors associated with Faraday cup-SEM cross gain calibration, as well as errors in the SEM dead-time measurements. After MC-ICP-MS measurements, all U/Th ages were calculated with the Isoplot/Ex 3.0 program [12], using decay constants of Cheng et al. [13]. Initial ^230^Th was corrected using an assumed bulk-Earth atomic ^230^Th/^232^Th ratio of 4.4±2.2 x 10^-6^, similar to the scheme used by Weisler et al. [9]. As most of the samples contain extremely low ^232^Th and thus have very high measured ^230^Th/^232^Th ratios, the initial ^230^Th correction has virtually no impact on the calculated ^230^Th ages. That is, there is nearly no difference between the corrected and uncorrected ^230^Th ages.

As the coral files might be collected live or dead, sub-aerially exposed, and then buried for a long time, the effect of secondary processes such as diagenesis on age accuracy was of vital concern. Fragmentation of the coral files into 1-2 mm grains followed by vigorous ultrasonic treatment in H_2_O_2_ proved to be very effective in removing organic matters and diagenetically generated materials (Figure S3) With the exception of samples 2011-024 and 2011-030, these considered to be the two freshest in the group, each coral abrader was dated twice, using materials from two separate 1-cm sections of each coral finger as described in Materials and Methods. If a coral finger has experienced diagenesis, it is unlikely that the diagenetic process would be homogeneous across the entire coral finger. In this regard, if the diagenetic influence was strong, then it would be expected that the U/Th dates derived from Sections A and B of the coral finger may differ significantly beyond their 2σ error range (Table S3). This is indeed the case for samples 2011-021, 027 and 028. For these samples their ages for Section A differ by 119-234 years, if compared with those for Section B. This is 5-10 times larger than the 2σ error ranges of the dates. This observation is further supported by the much dirtier appearance of these three samples if compared with the more pristine samples such as 2011-024 and 2011-036 (Figure S3). In the case of 2011-027, the sample looks significantly darker and dirtier after H_2_O_2_ treatment. The dates from this sample are considered the least reliable. The much higher ^232^Th is also evidence for significant diagenetic alteration and secondary Th addition. Except for the preceding three samples, which we exclude as unreliable, all other samples give consistent U/Th dates, as reflected by the ages of Sections A and B for each sample overlapping within their 2σ errors (Table S3). It is worthwhile to note that quite a number of samples contain high U, some up to 5.3 ppm, which is significantly higher than that of pristine corals (2-3.5 ppm). However, as illustrated by Yu et al. [14], higher than expected U contents were also found in the death assemblage of corals in the Great Barrier Reef that died over the last 100 years. This is attributable to microbialite-induced early diagenesis, occurring in many within 1-2 years of coral mortality [15]. As such diagenesis occurs so early, it has little impact on the age of the sample. Seawater origin of the elevated U in some corals is also evidenced by the fact that all the dated corals have their initial ^234^U/^238^U activity ratios clustering around the 2σ mean of 1.1463±0.0016, analytically indistinguishable from the mean value of 1.1468±0.0004 (2σ) for modern seawater [16]. The 2σ reproducibility of our data is larger than that of Andersen et al. [16] because our data were determined using the Faraday cup–electron multiplier (F-M) configuration designed for small sample sizes; those of Andersen et al. [16] were obtained using the multiple–Faraday cup (F-F) array designed for large sample sizes.

We are aware of the uncertainty and variability in the assumed value of 137.88 for the natural ^238^U/^235^U ratio [17]. However, ^238^U/^235^U values in most speleothem and coral materials are in the range of 137.7-137.8, similar to that for U-bearing minerals such as the international uraninite standard HU-1. As discussed above, in our MC-ICP-MS protocol, after every six unknown samples we measure an in-house standard that is calibrated against the ^234^U/^238^U and ^230^Th/^238^U values of the HU-1 standard assuming it is in secular equilibrium. The repeated measurements of the in-house standard are then used for external correction of any minor drift in the measured ^234^U/^238^U and ^230^Th/^238^U ratios of the samples. In addition, our detector configuration and analytical protocol allow the mass fractionation impacts on ^234^U/^238^U and ^230^Th/^238^U ratios to be cancelled out so that the measured ^230^Th/^234^U ratio, which is directly related to ^230^Th age, will not be affected by mass fractionation, or in other words, the choice of ^238^U/^235^U values for mass fractionation correction. Only the ^234^U/^238^U ratio, which has very little influence on the calculated ^230^Th age due to a much longer half life of ^234^U, will be affected by the choice of ^238^U/^235^U values for mass fractionation correction. Because of this, the age uncertainty arising from the assumed value for ^238^U/^235^U is minimized and negligible in most cases.

**Bibliography**

1. Poulsen J (1987) Early Tongan Prehistory, Terra Australis 12, Canberra: Australian National University. 2 vols.
2. Burley DV, Dickinson WR, Barton, A, Shutler R (2001), Lapita on the periphery, new data on old problems in the Kingdom of Tonga. Arch. in Oceania 36(2): 88-103.
3. Burley DV, Connaughton SP (2007) First Lapita settlement and its chronology in Vava’u, Kingdom of Tonga. Radiocarbon 49(1): 131-137.
4. Burley DV (1998) Tongan archaeology and the Tongan past - 2850 - 150 B.P. J. of World Preh. 12(3): 337-392.
5. Burley DV, Barton A, Dickinson WR, Connaughton SP, Taché K (2010) Nukuleka as a founder colony for West Polynesian settlement: New insights from recent excavations. J Pac Arch 1(2): 128-144.
6. Crabbe MJC, Smith DJ (2005) Sediment impacts on growth rates of Acropora and Porites corals from fringing reefs of Sulawesi, Indonesia. Coral Reefs 24: 437.
7. Zhao JX, Hu K. Collerson KD, Xu HK (2001) Thermal ionization mass spectrometry U-series dating of a hominid site near Nanjing, China. Geology 29(1): 27-30.
8. Hellstrom J (2003) Rapid and accurate U/Th dating using parallel ion-counting multi-collector ICP-MS. J. Analy. Atom. Spectr. 18(11): 1346-1351.
9. Weisler MI, Collerson KD, Feng Y, Zhao JX, Yu K (2006) Thoruim-230 coral chronology of a late prehistoric Hawaiian chiefdom. J Arch Sci 33: 273-282.
10. Clark TR, Zhao J-X, Feng Y-X, Done T, Jupiter SD, Lough JM, Pandolfi JM (2012) Spatial variability of initial ^230^Th/^232^Th in modern Porites from the inshore region of the Great Barrier Reef. Geochimica Et Cosmochimica Acta 78: 99-118.
11. Zhao JX, Hu K, Collerson KD, Xu HK (2001) 16]Thermal ionization mass spectrometry U-series dating of a hominid site near Nanjing, China. Geology 29(1): 27-30.
12. Ludwig KR (2003) Users Manual for Isoplot/Ex version 3.0: A Geochronological Toolkit for Microsoft Excel. In Berkeley Geochronology Centre Special Publication No.3
13. Cheng H, Edwards RL, Hoff J, Gallup CD, Richards DA, Asmerom Y (2000) The half-lives of uranium-234 and thorium-230. Chemical Geology 169(1-2): 17-33.
14. Yu KF, Zhao JX, Roff G, Lybolt M, Feng YX, Clark TR, Li S. (2012) High-precision U-series ages of transported coral blocks on Heron Reef (southern Great Barrier Reef) and storm activity during the past century. Palaeogeography Palaeoclimatology Palaeoecology 337–338: 23–36.
15. Nothdurft LD, Webb GE (2009) Earliest diagenesis in scleractinian coral skeletons: implications for palaeoclimate-sensitive geochemical archives. Facies 55: 161-201.
16. Andersen MB, Stirling CH, Zimmermann B, Halliday AN (2010) Precise determination of the open ocean ^234^U/^238^U composition. Geochemistry Geophysics Geosystems 11(12).
17. Stirling CH, Andersen MB., Potter EK, Halliday AN (2007) Low-temperature isotopic fractionation of uranium. Earth and Planetary Science Letters 264(1-2): 208-225.
18. Spennemann DHR, Head MJ (1998) Tongan pottery chronology, ^14^C dates and the hardwater effect. Quat Geochron 17: 1047-1056.
19. McCormac FG, Hogg AG, Blackwell PG, Buck CE, Higham TF, Reimer PJ (2004) ShCal04 southern hemisphere calibration, 0-11.0 kyr BP. Radiocarbon 46(3): 1087-1092.
